# Lack of efflux mediated quinolone resistance in *Salmonella enterica* serovars Typhi and Paratyphi A

**DOI:** 10.3389/fmicb.2014.00012

**Published:** 2014-01-27

**Authors:** Sylvie Baucheron, Isabelle Monchaux, Simon Le Hello, François-Xavier Weill, Axel Cloeckaert

**Affiliations:** ^1^UMR1282 Infectiologie et Santé Publique, Institut National de la Recherche AgronomiqueNouzilly, France; ^2^UMR1282 Infectiologie et Santé Publique, Université François Rabelais de ToursTours, France; ^3^Institut Pasteur, Unité des Bactéries Pathogènes Entériques, Centre National de Référence des Escherichia coli, Shigella et SalmonellaParis, France

**Keywords:** *Salmonella*, ciprofloxacin, transcriptional regulatory genes, *acrS*, efflux pumps

## Abstract

*Salmonella enterica* serovars Typhi and Paratyphi A isolates from human patients in France displaying different levels of resistance to quinolones or fluoroquinolones were studied for resistance mechanisms to these antimicrobial agents. All resistant isolates carried either single or multiple target gene mutations (i.e., in *gyrA*, *gyrB*, or *parC*) correlating with the resistance levels observed. Active efflux, through upregulation of multipartite efflux systems, has also been previously reported as contributing mechanism for other serovars. Therefore, we investigated also the occurrence of non-target gene mutations in regulatory regions affecting efflux pump expression. However, no mutation was detected in these regions in both Typhi and Paratyphi isolates of this study. Besides, no overexpression of the major efflux systems was observed for these isolates. Nevertheless, a large deletion of 2334 bp was identified in the *acrS*-*acrE* region of all *S*. Typhi strains but which did not affect the resistance phenotype. As being specific to *S.* Typhi, this deletion could be used for specific molecular detection purposes. In conclusion, the different levels of quinolone or FQ resistance in both *S*. Typhi and *S*. Paratyphi A seem to rely only on target modifications.

## Introduction

Enteric fever caused by the human-adapted pathogens *Salmonella enterica* serovars Typhi (*S*. Typhi) and Paratyphi A (*S*. Paratyphi A), B, and C, remains a major health problem (Crump and Mintz, 2010). A global epidemiologic study estimated that during the year 2000 typhoid fever caused 21.7 million illnesses and 21,7000 deaths and paratyphoid fever caused 5.4 million illnesses (Crump et al., 2004). During the past decade *S*. Paratyphi A was responsible for a growing proportion of enteric fever in Asia (Ochiai et al., 2005; Crump and Mintz, 2010). Enteric fever being associated with poor sanitation and unsafe food and water, it particularly affects children and adolescents in developing countries of Asia, Africa and Latin America (Crump et al., 2004; Bhan et al., 2005; Crump and Mintz, 2010). In developed countries, patients are most often ill-returned travellers or migrant workers (Bhan et al., 2005; Connor and Schwartz, 2005; Hassing et al., 2013).

To treat these infections, fluoroquinolones (FQ) and third-generation cephalosporins have been considered as first-line drugs, owing to the resistance to ampicillin, chloramphenicol, and trimethoprim/sulfamethoxazole that appeared during the 1980s (Hassing et al., 2011, 2013). Multidrug resistance (MDR) in *S*. Typhi is encoded mainly by resistance genes carried by large conjugative plasmids and has been reported worldwide (Le et al., 2007). As a consequence of a widespread FQ usage, *S*. Typhi and *S*. Paratyphi A isolates resistant to nalidixic acid (NAL^R^, minimum inhibitory concentration [MIC] > 16 mg/L) and with decreased susceptibility to ciprofloxacin (CIP^DS^, MIC 0.125–1.0 mg/L) have also emerged. Such NAL^R^-CIP^DS^ *S*. Typhi and *S*. Paratyphi A have been isolated in endemic areas and also in developed countries (Roumagnac et al., 2006; Le et al., 2007; Gaborieau et al., 2010; Accou-Demartin et al., 2011; Hassing et al., 2011, 2013).

Resistance to quinolones in *Salmonella* spp. is mostly attributed to point mutations in the quinolone resistance-determining regions (QRDRs) of the target genes *gyrA*, *gyrB*, *parC*, and *parE* (Cloeckaert and Chaslus-Dancla, 2001; Piddock, 2002; Velge et al., 2005; Giraud et al., 2006). For the *gyrA* gene, coding the A subunit of DNA gyrase, a single mutation resulting in an amino acid substitution at the position 83 (Serine to Phenylalanine or to Tyrosine) or at the position 87 (Aspartic acid to Asparagine or Glycine) has been the most frequently described in NAL^R^-CIP^DS^ *S*. Typhi and *S*. Paratyphi A isolates (Bhan et al., 2005; Renuka et al., 2005; Le et al., 2007; Gaborieau et al., 2010; Hassing et al., 2011). A second mutation leading to the amino acid change at the position 80 (Serine to Isoleucine or to Arginine) of the ParC subunit of topoisomerase IV was described to increase the CIP MIC (≥0.5 mg/L) in *S*. Typhi and *S*. Paratyphi A human isolates from India (Gaind et al., 2006). Whereas three mutations, i.e., a double mutation in *gyrA* at both codons 83 and 87 and one mutation in *parC*, were shown to confer CIP resistance (MIC >1 mg/L) in *S*. Typhi and *S*. Paratyphi A human isolates from India or from Taiwan (Gaind et al., 2006; Lee et al., 2013).

Moreover, the varying levels of CIP resistance observed in *S*. Typhi and *S*. Paratyphi A isolates with only a single *gyrA* mutation suggest that other mechanisms could be involved in quinolone resistance in this serovar (Renuka et al., 2005). Resistance to FQ in *S.* Typhimurium has also been attributed to active efflux mechanism, due to overproduction of the AcrAB-TolC efflux system (Giraud et al., 2000, 2006; Cloeckaert and Chaslus-Dancla, 2001; Piddock, 2006). We have previously reported the contribution of the AcrAB-TolC efflux system in resistance to FQ in several MDR epidemic clones of *S*. Typhimurium, such as *S*. Typhimurium of phage types DT204 or DT104 (Baucheron et al., 2002, 2004a,b). Among the chromosomal loci affecting AcrAB-TolC expression, the *ramRA* locus appears to be the most important in *Salmonella* spp. (Abouzeed et al., 2008; Kehrenberg et al., 2009). *ramR* encodes a repressor protein (RamR) belonging to the TetR family of repressor proteins, and has been shown to be the local repressor protein of *ramA* transcription (Abouzeed et al., 2008; Baucheron et al., 2012); while *ramA* encodes a transcriptional activator protein (RamA) belonging to the AraC/XylS family of regulatory proteins (Nikaido et al., 2008; Bailey et al., 2010). The latter is involved in upregulating expression of the AcrAB-TolC system (Nikaido et al., 2008; Bailey et al., 2010). Several mutations in *ramR* or its binding site upstream of *ramA*, affecting expression of this efflux system, have been detected in clinical isolates of serovar Typhimurium or Kentucky and of minor serovars Hadar, Infantis, Livingstone, or Schwarzengrund (Abouzeed et al., 2008; Kehrenberg et al., 2009; Hentschke et al., 2010; Akiyama and Khan, 2012; Baucheron et al., 2013).

In the present study, we have characterized mechanisms involved in resistance to quinolones or fluroquinolones in 21 *S*. Typhi and *S*. Paratyphi A strains displaying different levels of resistance to these drugs and isolated from patients in France during the period 1997–2008. For a subset of strains, with suspected increased efflux activity, we investigated the occurrence of mutations in the global *ram*, *sox* and *mar* regulatory loci of AcrAB-TolC, and in the local *acrR* and *acrS* repressor genes of the AcrAB and AcrEF pumps, respectively (Abouzeed et al., 2008; Kehrenberg et al., 2009).

## Materials and methods

### Bacterial strains

The twenty one strains including 16 *S*. Typhi and 5 *S*. Paratyphi A selected for this study were collected by the French National Reference Center for *Salmonella*, Institut Pasteur, Paris, France. They were isolated in France from travellers or migrants between 1997 and 2008 (Table 1). The selection was made to obtain diversity in terms of geographic origin, year of isolation, genetic lineages (haplotype for *S*. Typhi; Roumagnac et al., 2006), and phenotype of resistance to quinolones (Table 1).

**Table 1:** ***Salmonella enterica* serovars Typhi and Paratyphi A strains analyzed in this study**.

**Strain**	**Country**	**Year of isolation**	**Haplo type**	**Antimicrobial resistance pattern**	**MIC (mg/L)**		**Substitution(s) in the QRDR of:**				AcrA production ratio^*^
					**NAL**	**CIP**	**GyrA**	**GyrB**	**ParC**	**ParE**	
***SALMONELLA* TYPHI**											
06–423	India	2006	ND	Pansusceptible	4	0.015	WT	WT	WT	WT	1
06–426	India	2006	ND	CIP^DS^	16	0.125	WT	S464Y	WT	WT	1
02–1180	India	2002	H45	NALCIP^DS^	64	0.125	D87G	WT	WT	WT	1
05–3275	Morocco	2005	H6	NALCIP^DS^	64	0.125	D87N	WT	WT	WT	1
4(02)MB	Vietnam	1997	H58	ASCSulTmpTeNAL	128	0.03	S83Y	WT	WT	WT	0.5
222(97)MN	Vietnam	1996	ND	ASCSulTmpTeNALCIP^DS^	128	0.125	S83F	WT	WT	WT	0.5
43(97)MN	Vietnam	1996	H63	ASCSulTmpTeNALCIP^DS^	128	0.125	S83F	WT	WT	WT	0.5
98–3139	Mexico	1998	H50	NALCIP^DS^	128	0.125	S83F	WT	WT	WT	0.5
02–7744	India	2002	H52	NALCIP^DS^	128	0.125	S83F	WT	WT	WT	0.5
226(97)MN	Vietnam	1996	H61	ASCSulTmpTeNALCIP^DS^	128	0.25	S83F	WT	WT	WT	0.5
97–2307	India	1997	H63	NALCIP^DS^	256	0.125	S83F	WT	WT	WT	0.5
318(98)MB	Vietnam	1998	H58	ASCSulTmpTeNALCIP^DS^	512	0.25	S83Y	WT	WT	WT	1
39(98)MN	Vietnam	1998	H58	ASCSulTmpTeNALCIP^DS^	512	0.25	S83F	WT	WT	WT	1
4(02)MN	Vietnam	2000	H58	ASCSulTmpTeNALCIP^DS^	1024	0.25	S83F	WT	WT	D420N	1
5(04)MN	Vietnam	2004	ND	NALCIP^DS^	1024	0.25	S83F	WT	WT	D420N	1
04–2176	India	2004	H58	SSpSulTmpTeNALCIP	1024	8	S83F; D87N	WT	S80I	WT	1
***SALMONELLA* PARATYPHI A**											
08–8903	Senegal	2008		Pansusceptible	8	0.030	WT	WT	WT	WT	2
07–6329	Burkina Faso	2007		CIP^DS^	16	0.25	WT	S464F	WT	WT	2
05–208	India	2005		NALCIP^DS^	256	0.50	S83F	WT	WT	WT	2
08–4271	Guinea Bissau	2008		NACIP^DS^	1024	1	S83F	WT	WT	WT	2
08–2580	India	2008		NALCIP^DS^	1024	1	S83F	WT	WT	WT	3

* AcrA expression was measured by dot blotting with an anti-AcrA polyclonal antibody.

ND, not determined.

WT, wild type.

A, amoxicillin; S, streptomycin; Sp, spectinomycin; C, chloramphenicol; Sul, sulfamethoxazole; Tmp, trimethoprim; Te, tetracycline; NAL, nalidixic acid; CIP, ciprofloxacin; CIP^DS^, decreased susceptibility to ciprofloxacin.

D, aspartic acid; F, phenylalanine; G, glycine; I, isoleucine; N, asparagine; S, serine; Y, tyrosine.

### Antimicrobial susceptibility testing

Antimicrobial susceptibility was investigated by the standard disk diffusion method according to the recommendations of the Antibiogram Committee of the French Society for Microbiology (CA-SFM) (www.sfm-microbiologie.org/). The MICs of NAL and CIP were determined by the standard agar doubling dilution method as described previously (Baucheron et al., 2002). The NAL^R^ isolates were defined as having a MIC > 16 mg/L. The CIP^R^ isolates were defined as having a MIC > 1 mg/L and CIP^DS^ isolates as having a MIC comprised between 0.125 and 1.0 mg/L (Accou-Demartin et al., 2011; Hassing et al., 2013). MICs of these antibiotics were also determined in the presence of the efflux pump inhibitor Phe-Arg-β-naphthylamide (PAβ N, Sigma) at the following concentrations: 10, 20, 30, 40, 50, and 60 mg/L.

### Assessment of target-affecting mechanisms

Mutations in the QRDRs of *gyrA*, *gyrB*, *parC*, and *parE* genes were detected as described previously (Le et al., 2007; Song et al., 2010; Accou-Demartin et al., 2011).

The search of plasmid-mediated quinolone resistances genes, *qnrA*, *qnrB*, *qnrS*, *qnrD*, *aac*(6')-Ib-cr, and *qepA* was performed as described previously (Accou-Demartin et al., 2011).

### Assessment of efflux mechanisms

Efflux pump production was assessed by dot blot using an anti-AcrA polyclonal antibody as described previously (Abouzeed et al., 2008). Occurrence of mutations affecting *acrAB, acrEF*, and *tolC* expression was determined by PCR and sequencing the regulatory regions *ramR-ramA*, *acrR-acrA*, *marC*-marO-*marR-marA*, *soxS-soxR*, and *acrS-acrE* using primers listed in (Table 2). Transcription levels of efflux related genes *acrA*, *acrB*, *acrF*, *tolC*, *ramA*, and *ramR* were done by quantitative reverse transcription-PCR (qRT-PCR) as described previously (Baucheron et al., 2012; Giraud et al., 2013). Primers used for qRT-PCR are listed in (Table 2).

**Table 2:** **Primers used for PCRs**.

**Primer used and target region**	**Primer**	Nucleotide position relative to the LT2 strain genome sequence^*^	**Oligonucleotide sequence(s) (5'–3')**	**Size (bp)**	**Annealing temp (°C)**	**Reference**
**DETECTION OF MUTATIONS**						
*ramR-ramA*	ram5	638085	TCGGTAAAAGGCAGTTCCAG	958	60	Baucheron et al., 2013
	ramA6	639042	GTCGATAACCTGAGCGGAAA			
*acrR-acrA*	acrR1	533463	CAGTGGTTCCGTTTTTAGTG	992	58	Olliver et al., 2005
	acrR2	534454	ACAGAATAGCGACACAGAAA			
*marC*-marO*-marR-marA*	marR1	1597459	CAGTGTTGCGTCTGGACATC	787	60	Baucheron et al., 2013
	marR2	1598245	GCTAACGGGAGCAGTACGAC			
*soxS-soxR*	sox1	4503970	CTACAGGCGGTGACGGTAAT	915	60	Baucheron et al., 2013
	sox2	4504884	CGGCGCTTTAGTTTTAGGTG			
*acrS-acrE*	acrS3	3559106	AAAACGAACGGGAACTGATG	2874 ^***^	58	This study
	acrS4	3561978	ACAAACATACCGGGAAGCAG			
**qRT-PCR**						
*gmk*	gmk-f	3933294	TTGGCAGGGAGGCGTTT	62	60	Baucheron et al., 2012
	gmk-r	3933355	GCGCGAAGTGCCGTAGTAAT			
*gyrB*	gyrB-f	4040275	TCTCCTCACAGACCAAAGATAAGCT	81	60	Baucheron et al., 2012
	gyrB-r	4040195	CGCTCAGCAGTTCGTTCATC			
*rrs*	rrs-f	NA^**^	CCAGCAGCCGCGGTAAT	57	60	Baucheron et al., 2012
	rrs-r	NA^**^	TTTACGCCCAGTAATTCCGATT			
*ramA*	ramA-f	639180	GCGTGAACGGAAGCTAAAAC	167	60	Baucheron et al., 2012
	ramA-r	639346	GGCCATGCTTTTCTTTACGA			
*ramR*	ramR-f	638623	TAACGCAGGTGTTGCAGAAG	192	64	Baucheron et al., 2012
	ramR-r	638432	TGGTTCAGACCCCAACTGAT			
*acrA*	acrA-f	533120	GAAACCGCACGTATCAACCT	220	60	Baucheron et al., 2012
	acrA-r	532901	CCTGTTTCAGCGAACCATTT			
*acrB*	acrB-f	531348	TCGTGTTCCTGGTGATGTACCT	68	66	Baucheron et al., 2012
	acrB-r	531281	AACCGCAATAGTCGGAATCAA			
*acrF*	acrF-f	3563042	GCTCTGTCGTCCATCTCAAAGA	70	66	This study
	acrF-r	3563111	CGCGCTACAACGTTATAGTTTTCA			
*tolC*	tolC-f	3349107	GCCCGTGCGCAATATGAT	67	60	Baucheron et al., 2012
	tolC-r	3349173	CCGCGTTATCCAGGTTGTTG			

* GenBank NC_003197.1.

** NA: Not Applicable due to the number of copies of this gene in Salmonella.

**Figure 1:**
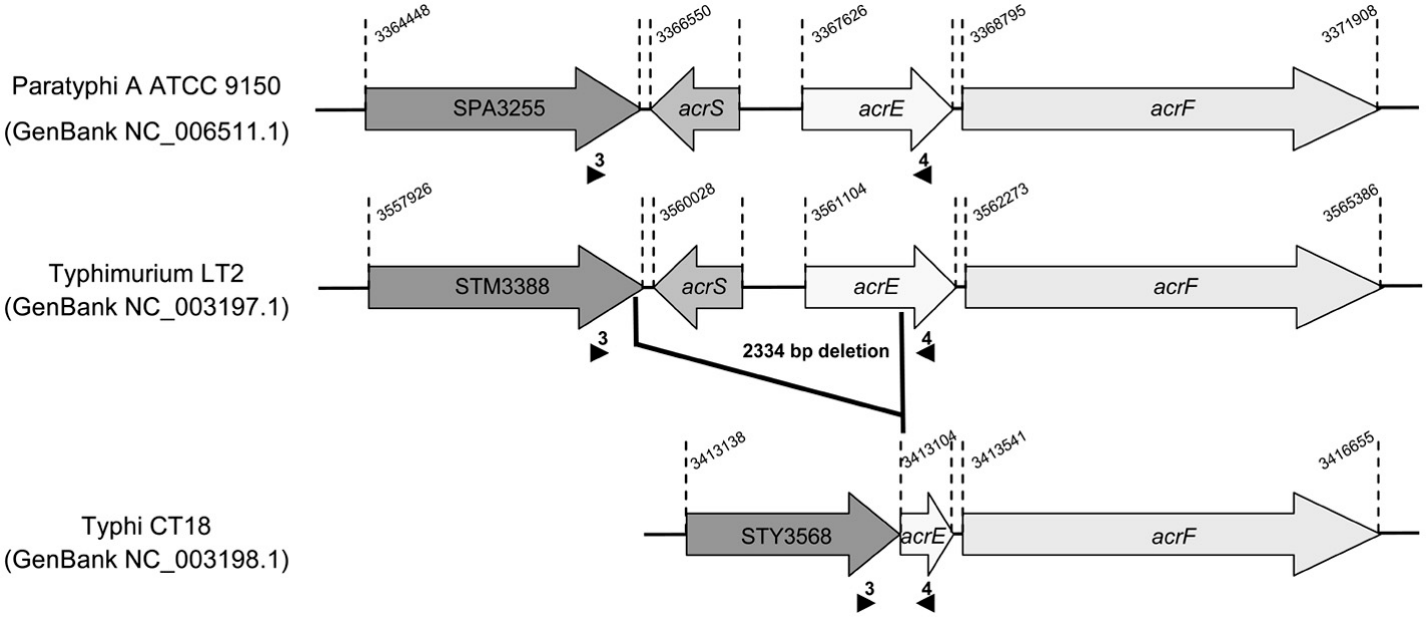
**Deletion identified in the *acrSE* region relative to the genome sequence of *S*. Typhi strain CT18 (GenBank NC_003198.1).** The sequence of the *acrSE* region of *S*. Typhi strain CT18 is compared to those of *S*. Paratyphi A strain ATCC9150 (GenBank NC_006511.1) and *S*. Typhi strain LT2 (GenBank NC_003197.1). Small black arrows indicate primers positions used for PCR to amplify and sequence the *acrSE* region. The 2334 bp chromosomal deletion was found in all *S*. Typhi strains studied.

*** 2874 bp for S. Typhimurium or S. Paratyphi A and 539 bp for S. Typhi (see Figure 1).

## Results and discussion

### Resistance phenotypes and target-affecting mechanisms

The twenty one *S*. Typhi and *S*. Paratyphi A strains of this study were isolated in France but acquired abroad, mainly in Asia and Africa (Table 1). Among the *S*. Typhi strains, all but two were NAL^R^ (MIC > 16 mg/L). One of the two strains was pansusceptible and the second was CIP^DS^ but only categorized as intermediate for NAL (NAL^I^, MIC 16 mg/L). Of the 14 NAL^R^ strains, one was CIP^*R*^, 12 were CIP^DS^ and one was CIP^S^ (MIC 0.03 mg/L). Eight NAL^R^ *S*. Typhi strains were also multidrug resistant. The majority of the NAL^R^ *S*. Typhi strains belonged to haplotype H58 which had emerged in Southern Asia during the mid 1990s (Roumagnac et al., 2006; Le et al., 2007). Among the *S*. Paratyphi A strains, all but two were NAL^R^. One of the two strains was pansusceptible and the second was NAL^I^-CIP^DS^. The three others were NAL^R^- CIP^DS^.

As shown in Table 1, both NAL^I^-CIP^DS^ had a mutation resulting in an amino acid substitution at position 464 of GyrB: serine to tyrosine for the *S*. Typhi isolate and serine to phenylalanine for the *S*. Paratyphi A isolate. The most frequent mechanism of resistance of NAL^R^-CIP^DS^ (*n* = 17) and NAL^R^-CIP^*S*^ (*n* = 1) strains, whatever the serovar, was a substitution at position 83 (serine to phenylalanine, *n* = 12, 66.6%) of GyrA. Other GyrA modifications were observed at position 83 (serine to tyrosine) in two isolates or at position 87 (aspartic acid to glycine, aspartic acid to asparagine) in one isolate for each. As described previously, a single substitution in GyrA was associated with resistance to nalidixic acid and decreased susceptibility to CIP (Bhan et al., 2005; Le et al., 2007; Gaborieau et al., 2010; Hassing et al., 2011). One exception was the *S*. Typhi strain 4 (02) MB, which was NAL^R^-CIP^S^ (and not CIP^DS^) despite a mutation in *gyrA* resulting in substitution serine to tyrosine at position 83.

Additional substitutions were found in ParE of 2 NAL^R^-CIP^DS^ *S*. Typhi strains that led to amino acid substitution aspartic acid to asparagine at position 420. In both cases, a 2-fold increase of NAL MICs was observed.

In the CIP^R^ *S*. Typhi isolate, three mutations leading to a double substitution in GyrA at positions 83 (serine to phenylalanine) and 87 (aspartic acid to asparagine) and one substitution at the position 80 of ParC (serine to isoleucine), as observed in previous studies (Renuka et al., 2005; Gaind et al., 2006; Lee et al., 2013).

The NAL^R^ and CIP^DS^ *S*. Typhi and *S*. Paratyphi A strains harboring a single substitution in GyrA showed various values for NAL (64–1024 mg/L) and CIP (0.03–0.5 mg/L) MICs which suggested the presence of other mechanisms of resistance. Since the plasmid-mediated quinolone resistance-conferring genes *qnrA*, *qnrB*, *qnrD* or *qnrS*, *qepA*, *and aac(6')-Ib-cr* were not detected, we investigated the role of the AcrAB-TolC efflux system.

### Involvement of efflux

None of the *S*. Typhi strains showed significant AcrA overproduction by dot blot, but nevertheless all *S*. Paratyphi A isolates showed a 2 or 3-fold increased AcrA production relative to the susceptible *S*. Typhi isolate (Table 1). Thus, overproduction of AcrA seems not to be involved in CIP^DS^ isolates compared with the susceptible isolates of *S*. Typhi or *S*. Paratyphi A. In presence of the efflux pump inhibitor PAβ N (20 or 40 mg/L), the CIP MICs similarly decreased (4 or 8-fold) in CIP^DS^ and in susceptible strains (Table 3 and data not shown), which is in accordance with previous studies on *S*. Typhimurium and corresponds to a decrease of resistance level observed for *acrB* or *tolC* deletion mutants (Baucheron et al., 2002, 2004b).

**Table 3:** **Study of efflux in a subset of *Salmonella enterica* serovars Typhi and Paratyphi A strains**.

**Strain**	**Antimicrobial resistance pattern^a^**	**MIC (mg/L)^b^**		**Substitution(s) in the QRDR of ^c^:**				***acrSE* sequencing**	**Transcription level of:**					
		**NAL**	**CIP**	**GyrA**	**GyrB**	**ParC**	**ParE**		***acrA***	***acrF***	***acrB***	***tolC***	***ramA***	***ramR***
***SALMONELLA* TYPHI**														
06–423	Pansusceptible	4	0.015 [0.004]	WT	WT	WT	WT	Deletion^d^	1.0	1.0	1.0	1.0	1.0	1.0
02–1180	NALCIP^DS^	64	0.125 [0.015]	D87G	WT	WT	WT	Deletion^d^	1.5	0.7	0.5	0.8	0.9	1.4
05–3275	NALCIP^DS^	64	0.125 [0.030]	D87N	WT	WT	WT	Deletion^d^	0.5	1.4	0.2	0.5	0.3	0.7
97–2307	NALCIP^DS^	256	0.125 [0.030]	S83F	WT	WT	WT	Deletion^d^	1.7	0.8	0.8	0.7	1.9	2.2
04–2176	SSpSulTmpTeNALCIP	1024	8 [2]	S83F; A87N	WT	S80I	WT	Deletion^d^	1.5	0.8	1.3	0.9	1.2	2.2
***SALMONELLA* PARATYPHI A**														
08–8903	Pansusceptible	8	0.030 [0.008]	WT	WT	WT	WT	WT	1.0	1.0	1.0	1.0	1.0	1.0
07–6329	CIP^DS^	16	0.25 [0.060]	WT	S464F	WT	WT	WT	1.6	1.4	1.0	1.0	1.0	1.2
05–208	NALCIP^DS^	256	0.50 [0.030]	S83F	WT	WT	WT	WT	1.3	0.9	1.4	1.0	1.1	1.0
08–4271	NALCIP^DS^	1024	1 [0.25]	S83F	WT	WT	WT	WT	1.2	1.1	0.7	1.4	1.3	1.3
08–2580	NALCIP^DS^	1024	1 [0.25]	S83F	WT	WT	WT	WT	2.0	1.6	1.4	1.4	2.0	1.3

a S, streptomycin; Sp, spectinomycin; Sul, sulfamethoxazole; Tmp, trimethoprim; Te, tetracycline; NAL, nalidixic acid; CIP, ciprofloxacin; CIP^DS^, decreased susceptibility to ciprofloxacin.

b Values in brackets are MICs in the presence of the efflux pump inhibitor PAβ N at 40 mg/L.

c WT, wild type; D, aspartic acid; F, phenylalanine; G, glycine; I, isoleucine; N, asparagine; S, serine.

d 2334 bp deleted.

Despite a lack of evidence of increased efflux in the resistance phenotype, we measured by qRT-PCR the transcription levels of efflux related genes *acrA*, *acrF*, *acrB*, *tolC*, *ramA*, and *ramR* in CIP^DS^ non-MDR strains and in the CIP^R^ strain. No differences were detected in the transcription levels of these genes, between susceptible, CIP^DS^ and CIP^R^ strains, whatever the serovar (Table 3). In addition, no mutations were detected in the regulatory regions of the AcrAB-TolC efflux system. However, during the screening of the regulatory regions, we identified a single large deletion of 2334 bp in the *acrS*-*acrE* region of all *S*. Typhi strains, including the susceptible one (Table 3). This deletion encompassed the *acrS* gene, that encodes a transcriptional repressor, and a large part of the *acrE* gene that encodes the AcrE periplasmic lipoprotein, which is homologous to AcrA (Olliver et al., 2005). This 2334 bp deletion was also observed in the *acrS*-*acrE* region of sequenced genomes of MDR *S*. Typhi CT18 strain (Parkhill et al., 2001) and pansusceptible TY2 strain (Deng et al., 2003) (Figure 1). Previously, it has been shown that *acrS* deletion in *S*. Typhimurium does not affect *acrEF* expression (Olliver et al., 2005). Similarly the natural *acrSE* deletion detected in *S*. Typhi had no impact on the *acrF* transcription level as observed in this study. To our knowledge, this is the first description of such a natural *acrS*-*acrE* chromosomal deletion and seems specific to *S*. Typhi since it was not detected in all currently sequenced genomes of the other serovars (not shown).

## Conclusions

The main mechanisms involved in quinolone or FQ resistance in both *S*. Typhi and *S*. Paratyphi A are target modifications. In contrast to what is seen in enteric pathogenic serovars, such as Typhimurium or the emerging CIP^R^ Kentucky ST198 clone (Baucheron et al., 2013), increased efflux pump production-mediated mechanisms seem to be totally absent in both *S*. Typhi and *S*. Paratyphi A. The deletion identified in the *acrSEF* region, although not involved in the resistance phenotype, may be helpful for the specific detection of *S*. Typhi.

### Conflict of interest statement

The authors declare that the research was conducted in the absence of any commercial or financial relationships that could be construed as a potential conflict of interest.
